# *RUNX1* Is a Key Target in t(4;11) Leukemias that Contributes to Gene Activation through an AF4-MLL Complex Interaction

**DOI:** 10.1016/j.celrep.2012.12.016

**Published:** 2013-01-31

**Authors:** Adam C. Wilkinson, Erica Ballabio, Huimin Geng, Phillip North, Marta Tapia, Jon Kerry, Debabrata Biswas, Robert G. Roeder, C. David Allis, Ari Melnick, Marella F.T.R. de Bruijn, Thomas A. Milne

**Affiliations:** 1 MRC Molecular Haematology Unit, Weatherall Institute of Molecular Medicine, University of Oxford, Oxford OX3 9DS, UK; 2 Departments of Medicine/Hematology and Oncology Division, Weill Medical College of Cornell University, New York, NY, 10065, USA; 3 Department of Pharmacology, Weill Medical College of Cornell University, New York, NY, 10065, USA; 4 Institute for Computational Biomedicine, Weill Medical College of Cornell University, New York, NY, 10065, USA; 5 Department of Laboratory Medicine, University of California, San Francisco, San Francisco, CA 94143, USA; 6 Laboratory of Biochemistry and Molecular Biology, The Rockefeller University, New York, NY 10065, USA; 7 Laboratory of Chromatin Biology and Epigenetics, The Rockefeller University, New York, NY 10065, USA

## Abstract

The Mixed Lineage Leukemia (MLL) protein is an important epigenetic regulator required for the maintenance of gene activation during development. *MLL* chromosomal translocations produce novel fusion proteins that cause aggressive leukemias in humans. Individual MLL fusion proteins have distinct leukemic phenotypes even when expressed in the same cell type, but how this distinction is delineated on a molecular level is poorly understood. Here, we highlight a unique molecular mechanism whereby the *RUNX1* gene is directly activated by MLL-AF4 and the RUNX1 protein interacts with the product of the reciprocal AF4-MLL translocation. These results support a mechanism of transformation whereby two oncogenic fusion proteins cooperate by activating a target gene and then modulating the function of its downstream product.

## Graphical abstract

**Graphical Abstract:**

**Figure:**
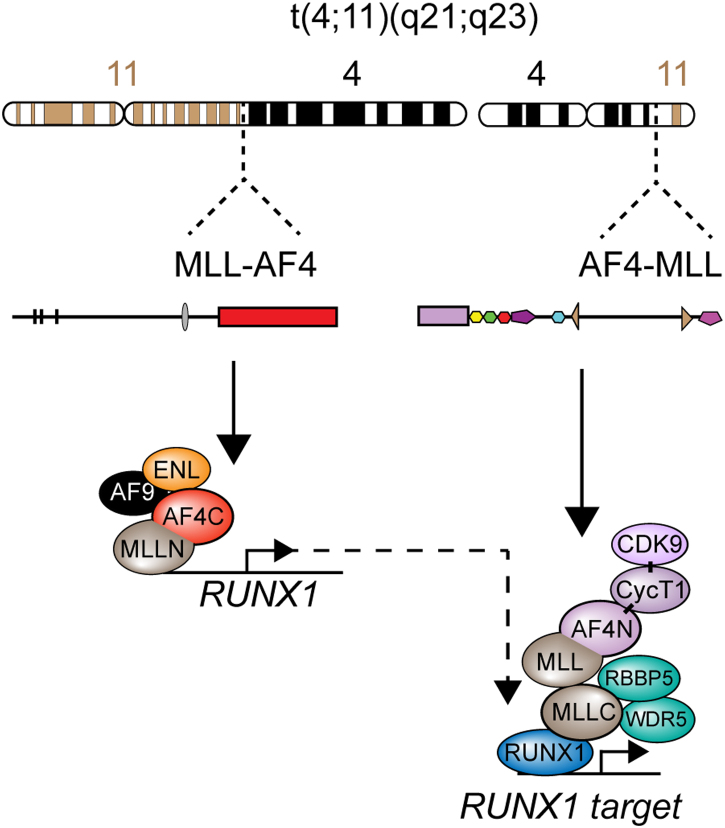

**Highlights:**

► A common set of target genes directly regulated by MLL-AF4 is identified ► *RUNX1* is a target gene that is specifically upregulated in t(4;11) patients ► MLL-AF4 controls *RUNX1* gene expression by stabilizing ENL and AF9 binding ► RUNX1 cooperates with an AF4-MLL complex to activate gene targets

## Teaser

Children and adults with acute leukemias caused by the mixed lineage leukemia (*MLL*) gene have very poor survival rates, most likely due to the fact that MLL is a regulator of epigenetic information. Milne and colleagues now show that a protein named RUNX1 cooperates with two different *MLL* mutations to alter the epigenetic information content of the cell, directly contributing to the poor prognosis of MLL-associated leukemias.

## Introduction

Aberrant epigenetic changes are a driving force in many cancers and are excellent candidates for the development of targeted therapies (Dawson and Kouzarides, 2012). The design of such therapies depends on a clear understanding of the molecular details of disease progression. The Mixed Lineage Leukemia (MLL) protein is an example of an important epigenetic protein that is mutated in a subset of aggressive leukemias (Marschalek, 2010), and thus provides a useful model for studying the link between epigenetic changes and cancer progression.

MLL is important for the epigenetic maintenance of gene activation and is required for normal hematopoietic development (Jude et al., 2007; McMahon et al., 2007). *MLL* leukemogenic mutations include chromosomal translocations (commonly called *MLL* rearrangements [MLLr]) that fuse the N terminus of the *MLL* gene in-frame with any one of over 60 different partner genes, producing novel fusion proteins (MLL-FPs). Almost 90% of all MLL-FPs are fusions with AF4, AF9, ENL, ELL, AF10, or AF6 (Meyer et al., 2009).

t(4;11)(q21;q23) chromosome translocations (referred to from this point as t(4;11) translocations) fuse *MLL* in-frame with the *AF4* gene and produce both MLL-AF4 and AF4-MLL fusion proteins (Bursen et al., 2004; Bursen et al., 2010). t(4;11) translocations are a major cause of infant acute lymphoblastic leukemia (ALL) and produce an aggressive disease with a poor prognosis. Enforced expression of MLL-AF4 alone is incapable of transforming human CD34^+^ cord blood (Montes et al., 2011), and mouse models expressing MLL-AF4 alone are not fully representative of the human disease, instead producing B-cell lymphomas (Chen et al., 2006; Metzler et al., 2006) acute myeloid leukemia (AML), or precursor B-ALLs (pre-B-ALLs) (Krivtsov et al., 2008). Conversely, expression of both MLL-AF4 and AF4-MLL together result in either common lymphoid progenitor leukemia or mixed lineage leukemia (MLL), a close recapitulation of the human disease (Bursen et al., 2010). Unlike many acute leukemias, t(4;11) leukemias are associated with very few cooperating mutations (Bardini et al., 2010, 2011). This suggests that the t(4;11) translocation by itself may be sufficient for leukemic transformation, perhaps because both MLL-AF4 and AF4-MLL fusion proteins are capable of altering the epigenetic information content of the cell (Benedikt et al., 2011; Krivtsov et al., 2008). Interestingly, knocking down the MLL-AF4 fusion protein alone is sufficient to disrupt t(4;11) leukemic growth in vivo (Thomas et al., 2005), indicating that targeting pathways controlled by the MLL-AF4 protein could be effective in treating t(4;11) leukemias.

Wild-type MLL is proteolytically cleaved in vivo by Taspase 1 into N-terminal (MLL-N) and C-terminal (MLL-C) proteins, which then dimerize in the presence of a large protein complex (Dou et al., 2005; Hsieh et al., 2003; Nakamura et al., 2002; Yokoyama et al., 2002). MLL normally maintains activation of target genes through multiple epigenetic mechanisms including the trimethylation of histone 3 on lysine 4 (H3K4Me3) via the activity of its C-terminal SET domain, and through the recruitment of transcriptional coactivators such as RBBP5, WDR5, ASH2L, and the H4 acetyltransferase MOF (Dou et al., 2005; Milne et al., 2002; Nakamura et al., 2002). MLL-FPs lack the C-terminal SET domain, and five of the most common MLL-FPs are constituents of a large interactome that includes the transcriptional coactivator complex pTEFb (a dimer of Cyclin T1 and CDK9; Marshall and Price, 1995) and other members of a “super elongation complex” (SEC) (Biswas et al., 2011; Lin et al., 2010; Mueller et al., 2007; Yokoyama et al., 2010; Zeisig et al., 2005), the H3K79 methyltransferase DOT1L (Biswas et al., 2011; Okada et al., 2005), the histone acetyl interacting bromodomain-containing protein 4 (BRD4, a member of the BET family of bromodomain proteins) (Dawson et al., 2011; Zuber et al., 2011), as well as the polymerase associated factor 1 (PAF1) complex (Milne et al., 2010; Muntean et al., 2010). The current model of MLL-FP function implicates BRD4 and PAF1 in recruitment of MLL-FPs and the SEC to a subset of important target genes causing increased transcription elongation (Biswas et al., 2011; Dawson et al., 2011; Lin et al., 2010; Milne et al., 2010; Mueller et al., 2007; Muntean et al., 2010; Yokoyama et al., 2010; Zuber et al., 2011). Interestingly, AF4-MLL copurifies with a pTEFb-containing complex, and is thought to induce gene activation through a similar SEC recruitment mechanism (Benedikt et al., 2011).

Although work with BRD4 inhibitors suggests that multiple MLL-FPs use the same molecular pathway for leukemogenesis (Dawson et al., 2011; Zuber et al., 2011), this cannot explain the fact that MLL-AF4, MLL-AF9, and MLL-ENL produce different leukemias even when expressed in the same cell type (Drynan et al., 2005; Metzler et al., 2006). Furthermore, gene expression analyses in t(4;11), MLL-ENL, and MLL-AF9 patient samples display overlapping as well as distinct gene expression profiles (Stam et al., 2010; Trentin et al., 2009), indicating that individual MLL-FPs could activate unique gene expression pathways.

In this study, we set out to further analyze t(4;11) leukemias on a molecular level. We initially used chromatin immunoprecipitation sequencing (ChIP-seq) and MLL-AF4 small interfering RNAs (siRNAs) in patient cell lines to identify key gene targets regulated by MLL-AF4. One direct target of the MLL-AF4 protein is the *RUNX1* gene, a key hematopoietic transcription factor that is specifically overexpressed in t(4;11) patient samples. Distinct from other MLL-FPs, *RUNX1* expression is important for the growth of t(4;11) leukemia cell lines, in which it plays a role in the activation of specific target genes. Furthermore, we show that RUNX1 interacts with an AF4-MLL complex, providing a new model of how MLL-AF4 and AF4-MLL cooperate on a molecular level. Such a cooperative effect between these two fusion proteins could explain some of the differences between t(4;11) and other MLL-FP leukemias.

## Results

### Common MLL-AF4 Target Genes Are Overexpressed in Primary B-ALL Patient Samples

To identify potentially important direct target genes of MLL-AF4 in t(4;11) leukemias, we performed ChIP-seq in the RS4;11 cell line and compared our results to a previously published data set from SEM cells (Guenther et al., 2008). RS4;11 and SEM cell lines are both t(4;11) pre-B-ALL patient-derived cell lines (see Extended Experimental Procedures for details on cell lines) that express the MLL-AF4 protein as well as wild-type MLL and wild-type AF4.

**Figure 1:**
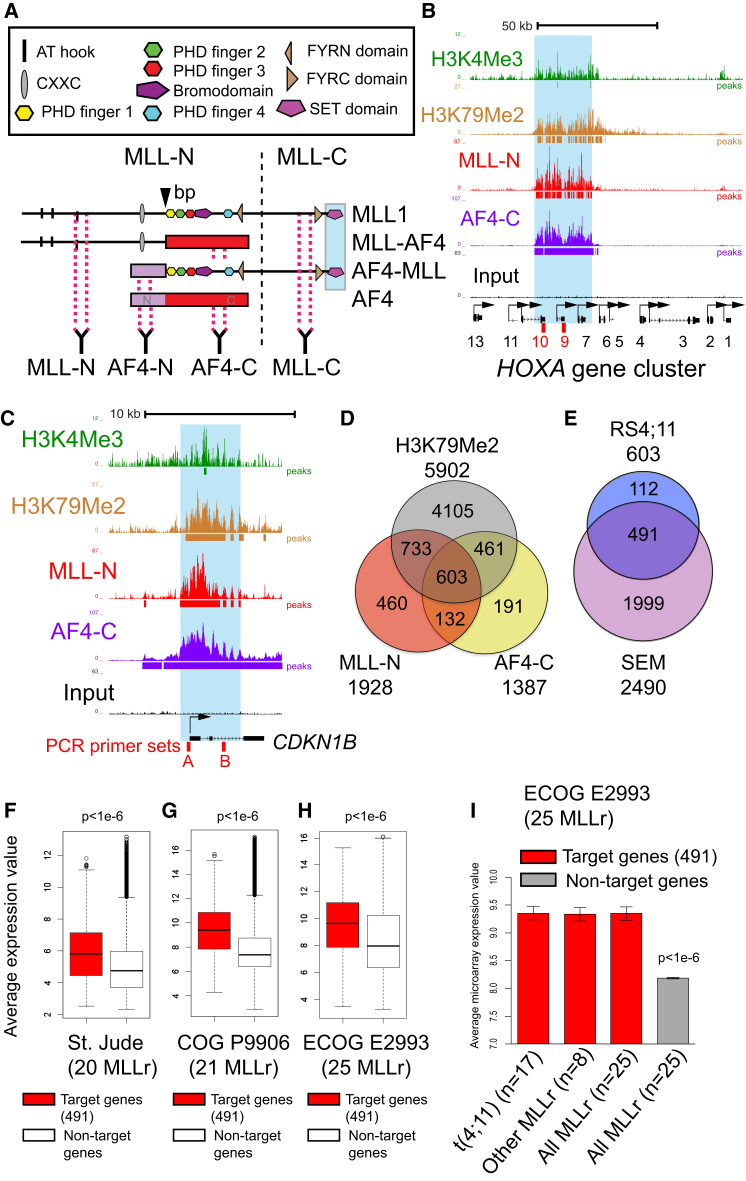
MLL-AF4 ChIP-Seq Target Genes Are Upregulated in Primary B-ALLs (A) Wild-type MLL is proteolytically cleaved (dashed line) into N-terminal (MLL-N) and C-terminal (MLL-C) proteins. The t(4;11) breakpoint is marked by a black arrowhead labeled “bp.” The translocation fuses part of MLL-N in-frame with AF4-C (red box), and also produces a reciprocal AF4-MLL fusing AF4-N (violet box) with the rest of MLL. Antibody positions on the wild-type and fusion proteins are shown. A RUNX1 interaction domain at the C-terminal SET domain (Huang et al., 2011) is indicated by blue shading. (B and C) ChIP-seq in RS4;11 cells across the *HOXA* cluster (B) and *CDKN1B* (C). The number of reads for peak summits was normalized by the total number of reads per track (set to 1 Gb for each track). Four different primer sets used for real-time PCR ChIP analysis are shown (red boxes) for the following amplicons: *A9*, *A10*, *CDKN1B*-A, and -B. (D) ChIP-seq in RS4;11 cells using antibodies to MLL-N, AF4-C, and H3K79Me2 produced an overlap at 603 target genes. (E) Comparison between the 603 RS4;11 target gene set from (D) and similar ChIP-seq data from SEM cells (Guenther et al., 2008) produced a set of 491 common MLL-AF4 targets (see Table S1). (F–I) The average expression of the 491 MLL-AF4 fusion target genes common in RS4;11 and SEM cells have significantly higher (p < 1e-6, two-tailed Wilcoxon test) expression levels than the nontarget genes in MLLr B-ALL patients in three different B-ALL clinical trials. (F) St. Jude Children’s Research Hospital, n = 20 MLLr patients (Ross et al., 2003). (G) COG P9906 clinical trial, n = 21 MLLr patients (Harvey et al., 2010). (H) ECOG E2993 clinical trial, n = 25 MLLr patients (Geng et al., 2012). (I) The same data as in (H), split into t(4;11) versus other MLLr patient samples. Boxplots (F–H) represent the median values and error bars represent extreme maximum and minimum whisker values for each plot. Bar plots (I) are the mean and error bars represent SEM. See also Table S1 and Figure S1.

**Figure S1:**
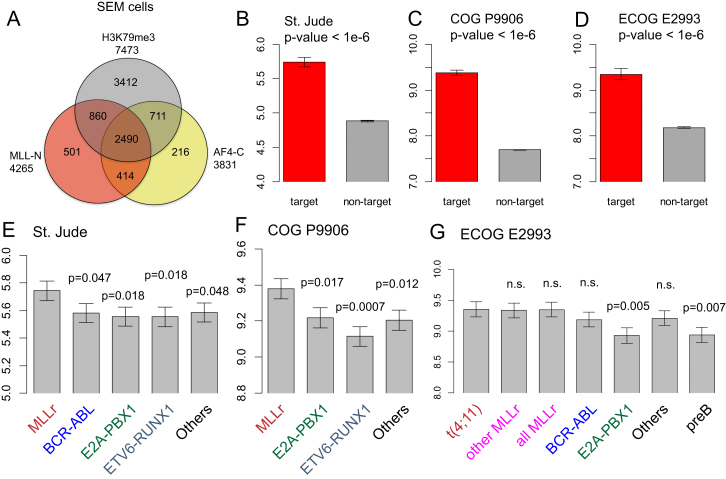
MLL-AF4 Targets Genes in SEM Cells and Expression in ALL Patient Samples, Related to Figure 1 (A) MLL-AF4 target genes in SEM cells. ChIPseq analysis in SEM cells with antibodies to MLL-N, AF4-C and H3K79Me2 using data from (Guenther et al., 2008) produced an overlap of 2490 target genes (list in Table S1). (B–D) Average expression of the 491 target gene set (and the non target gene set) in MLL rearranged (MLLr) B-ALL patients from three B-ALL clinical trials expressed as bar plots with the error bars representing s.e.m. (standard error of mean). (B) St. Jude Children’s Research Hospital, n = 132 with 20 MLLr patients (Ross et al., 2003). (C) COG P9906 clinical trial, n = 207 with 21 MLLr patients (Harvey et al., 2010). (D) ECOG E2993 clinical trial, n = 191 with 25 MLLr patients (Geng et al., 2012). (E–G) Average expression 491 target gene set expressed as barplots with the error bars representing s.e.m in clinical trials from St. Jude Children’s Research Hospital, BCR-ABL patients: 15, E2A-PBX1 patients: 18, MLLr patients: 20, RUNX1-ETV6 patients: 20, Other B-ALL patients: 28 (E), COG P9906 clinical trial, E2A-PBX1 patients: 23, MLLr patients: 21, RUNX1-ETV6 patients: 3, Other B-ALL patients: 155, (F) ECOG E2993 clinical trial, BCR-ABL patients: 78, E2A-PBX1 patients: 6, MLLr patients: 25 (t(4;11): 17, other MLLr: 8, Other B-ALL patients: 82.

No single antibody has been developed to uniquely recognize endogenous MLL-AF4. Instead, using an approach originally taken by Guenther et al. (2008), we performed ChIP-seq experiments using antibodies against the N terminus of MLL (αMLL-N) and the C terminus of AF4 (αAF4-C) (Figure 1A). To find actively transcribed gene targets bound by MLL-AF4, we identified promoters in RS4;11 cells enriched for both MLL-N and AF4-C as well as H3K79Me2 (an active transcription elongation mark that is highly enriched at important MLL-FP target genes; Krivtsov et al., 2008; Milne et al., 2005) enrichment within the gene body (Figures 1B–1D). We identified 603 gene targets that met all three criteria (Figure 1D; Table S1). Two previously identified direct targets of MLL-FPs, the *HOXA* cluster (Bernt et al., 2011; Guenther et al., 2008; Milne et al., 2005) and *CDKN1B* (Bernt et al., 2011; Xia et al., 2005), are shown as examples (Figure 1B and C). A similar approach with the SEM cell ChIP-seq data set (Guenther et al., 2008) identified 2,490 putative MLL-AF4 target genes (Figure S1A; Table S1), which produced a common overlap of 491 genes (Figure 1E; Table S1). The 491 target set includes previously identified targets such as *JMJD1C, BCL2*, *FLT3*, *MYB*, *MYC*, *RUNX2*, *MEIS1, CDKN1B,* and *HOXA* cluster genes, as well as some other potentially interesting gene targets such as *EZH2*, *FOXP1*, *IZKF1*, *IZKF2,* and *SOX4* (Table S1).

In MLLr B-ALL patient samples from three large clinical studies, the average expression of the 491 MLL-AF4 target genes was significantly higher than that of nontarget genes (Figures 1F–1H and S1B–S1D). The 491 target gene set is also significantly overexpressed in MLLr ALL compared to several other B-ALL subtypes (E2A-PBX1, ETV6-RUNX1, and pre-B; Figure S1E–S1G), although not others (e.g., BCR-ABL; Figure S1G). There is no significant difference between t(4;11) and other MLLr patient samples (Figure 1I), suggesting that this 491 gene target set is generally overexpressed in patients with MLLr ALLs. This correlation between ChIP-seq and gene expression data in patient samples validates ChIP-seq in patient cell lines as a powerful method to identify important target genes, and also suggests that our 491 common MLL-AF4 targets have an in vivo relevance to MLLr leukemia in human patients.

### *RUNX1* Is Overexpressed in Primary ALLs with t(4;11)

High expression of *HOXA9*, *HOXA10,* and *MEIS1* is considered to be a general hallmark of all MLL-FP leukemias, but a detailed analysis of patient expression data show that many t(4;11) leukemias do not express high levels of these genes (Stam et al., 2010; Trentin et al., 2009), indicating that other additional targets are likely to have an important role in t(4;11) leukemogenesis.

**Figure 2:**
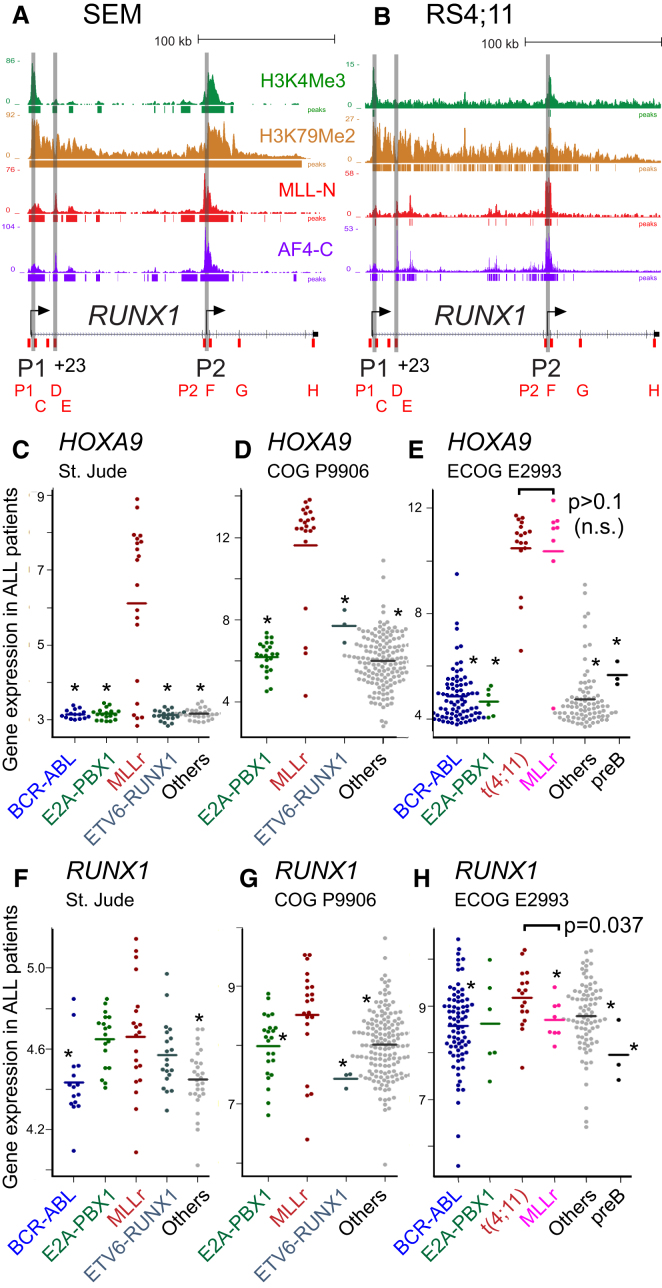
*RUNX1* Is a Direct Target of MLL-AF4 and Is Specifically Upregulated in t(4;11) B-ALLs (A and B) ChIP-seq data in SEM (A) and in RS4;11 (B) cells across the *RUNX1* locus using the antibodies as indicated. Reads were normalized as in Figure 1. Gray bars highlight the positions of the P1 and P2 promoters as well as the +23 enhancer. Primer sets used for real-time PCR ChIP analysis are shown (red boxes). (C–H) The average expression of either *HOXA9* (C–E) or *RUNX1* (F–H) in three B-ALL clinical trials separated into different ALL subtypes as indicated. (C and F) St. Jude ALL patients (Ross et al., 2003). (D and G) COG P9906 clinical trial (Harvey et al., 2010). (E and H) ECOG E2993 clinical trial (Geng et al., 2012). An asterisk indicates significantly lower average expression for the leukemia subtype relative to MLLr (C, D, F, and G) or relative to t(4;11) (E and H). A two-tailed Wilcoxon test was used to calculate p values, and p values for the different comparisons are in Table S2. See also Tables S3, S4, and Figure S2.

**Figure S2:**
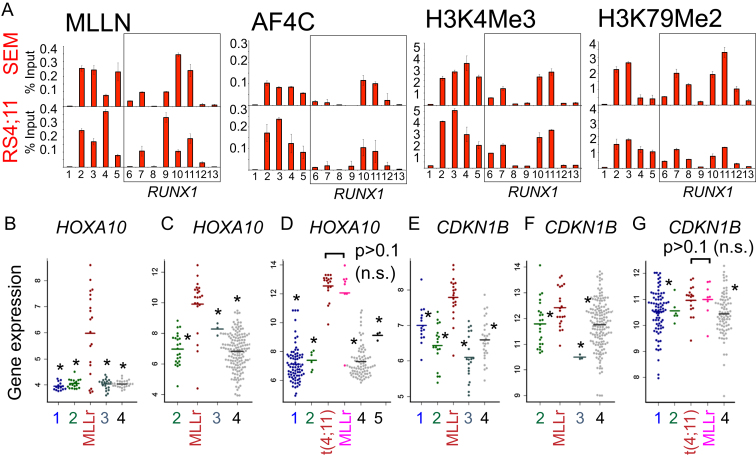
ChIP across *RUNX1* and Additional Gene Expression in ALL Patients, Related to Figure 2 (A) MLL-N, H3K4Me3 and H3K79Me2 ChIP in SEM (upper bar plots) and RS4;11 (lower bar plots) cells at a control region (1), *HOXA9* (2), *HOXA10* (3), *CDKN1B* (4 and 5), and across *RUNX1* (6-13). Primer sets are as explained in Figures 1 and 2. (B–G) *HOXA10* and *CDKN1B* are upregulated in primary B-ALLs with *MLL1* rearrangements (MLLr). The average expression of either *HOXA10* (B–D) or *CDKN1B* (E–G) was examined in B-ALL subtypes (including several non-MLL fusion proteins) using data from patients participating in 3 large B-ALL clinical trials. (B) and (E) St. Jude Children’s Research Hospital, (Ross et al., 2003) separated into the following subtypes: BCR-ABL (blue 1), n = 15; E2A-PBX1 (green 2), n = 18; MLL rearrangements (MLLr), n = 20; ETV6-RUNX1 (teal 3), n = 20; Others (black 4), n = 28. (C) and (F) COG P9906 clinical trial, (Harvey et al., 2010) separated into the following subtypes: E2A-PBX1 (green 2), n = 23; MLLr, n = 21; ETV6/RUNX1 (teal 3), n = 3; Others (black 4), n = 155. (D) and (G) ECOG E2993 clinical trial, (Geng et al., 2012) separated into the following subtypes: BCR-ABL (blue 1), n = 78; E2A-PBX1 (green 2), n = 6; t(4;11), n = 17; Other MLLr, n = 8; Others (black 4), n = 82; normal preB cells (black 5), n = 3. An asterisk indicates significantly lower average expression for the leukemia subtype relative to MLLr (B,C,E,F) or relative to MLL-AF4 (D,G). *HOXA10* and *CDKN1B* expression is not significantly different in MLLr versus MLL-AF4 samples (F and I, p > 0.1). Other p values for the different comparisons are in Table S2.

Among our list of 491 potential MLL-AF4 target genes, the master hematopoietic transcription factor *RUNX1* (*AML1*) is highly enriched for MLL-N, AF4-C, H3K79Me2, and H3K4Me3 in RS4;11 and also in SEM cells, at both of its two promoters, and at the hematopoietic +23 enhancer element (Nottingham et al., 2007) (Figures 2A, 2B, and S2A). Although Guenther et al. (2008) used different specific antibodies than those used in this study, a direct comparison using our own antibodies in conventional ChIP experiments suggests that RS4;11 and SEM cells have similar levels of MLL-N, AF4-C, H3K4Me3, and H3K79Me2 enrichment across *RUNX1* (Figure S2A).

Mutations in *RUNX1* are commonly associated with AML but are also found in B-ALL and T-ALL, and are usually inactivating, suggesting that RUNX1 normally functions as a tumor suppressor (Blyth et al., 2005; Mangan and Speck, 2011; Zhang et al., 2012). However, overexpression of wild-type *RUNX1* can be oncogenic (Blyth et al., 2005). Thus, considering the crucial role of RUNX1 in hematopoiesis and many acute leukemias, we decided to further explore its potential role in t(4;11) leukemias.

We analyzed the expression of *RUNX1* and other target genes in specific subsets of primary ALL samples, including t(4;11) and other MLLr samples. Average *HOXA9*, *HOXA10,* and *CDKN1B* expression is significantly higher in MLLr leukemias than in other ALL subtypes (Figures 2C–2E and S2B–S2G; Table S2), but no significant difference in expression levels is seen when comparing MLLr and t(4;11) leukemias (in the Eastern Cooperative Oncology Group [ECOG] E2993 patient set, the only set where this MLLr cytogenetic information is available; Figures 2E, S2D, and S2G; Table S2). Interestingly, similar to what has been reported previously (Stam et al., 2010; Trentin et al., 2009), several individual patients display relatively low expression of both *HOXA9* and *HOXA10* (Figures 2C–2E and S2B–S2D).

With the exception of E2A-PBX1 leukemias, *RUNX1* is significantly overexpressed in MLLr leukemias compared to other ALL subtypes (Figures 2F–2H; Table S2). Importantly, in the ECOG E2993 patient set, *RUNX1* is significantly overexpressed in t(4;11) samples compared to the other MLLr samples (Figure 2H; Table S2). Interestingly, the non-t(4;11) MLLr samples in the ECOG E2993 data set appear to have a lower than average expression of *RUNX1* compared to other leukemia subtypes (Figure 2H). One possibility is that t(4;11) samples account for the bulk of the high-expressing *RUNX1* samples in the St. Jude and Children’s Oncology Group (COG) P9906 data sets (Figures 2F and 2G), but unfortunately, because we do not have t(4;11)-specific data on individual MLLr samples in these data sets, we cannot test this directly.

These results are also consistent with a recent analysis that showed *RUNX1* is specifically overexpressed in t(4;11) samples compared to several other childhood ALL samples (Montero-Ruíz et al., 2012). It is worth pointing out that the statistically significant increase in *RUNX1* expression in the ECOG E2993 data set only represents a 1.3- to 2.3-fold change in microarray expression (Tables S3 and S4). However, a small change in messenger RNA levels for an important master regulatory protein such as RUNX1 could represent a much bigger effect at the protein level. Taken as a whole, these results suggest the possibility that *RUNX1* could have a unique role in t(4;11)-mediated leukemogenesis, and we therefore decided to analyze its possible role on a more detailed molecular level.

### MLL-AF4 Directly Regulates *RUNX1* and Other Target Loci by Stabilizing ENL and AF9 Binding

**Figure 3:**
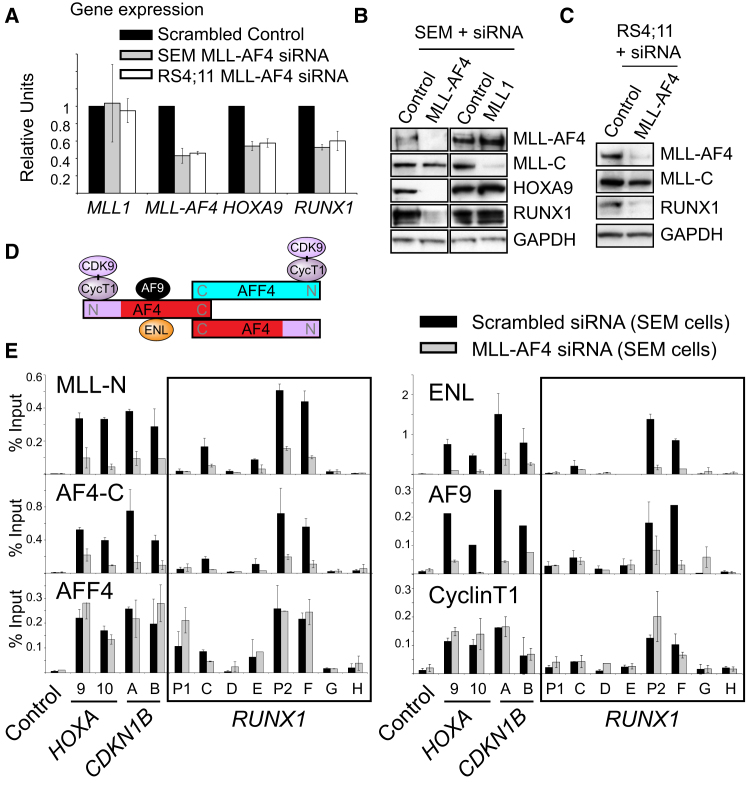
MLL-AF4 Directly Regulates *RUNX1* and Other Target Genes by Stabilizing AF9 and ENL Binding (A) *MLL*, *MLL-AF4*, *HOXA9,* and *RUNX1* real-time PCR expression in scrambled control siRNA-treated cells (black bars), MLL-AF4 siRNA-treated SEM (gray bars), and RS4;11 (white bars) cells. Data are the mean ± SD (error bars) of three independent knockdown experiments. In each individual experiment, control values were set to 1. (B and C) Western blots as indicated in SEM cells (B) or RS4;11 cells (C) treated with the siRNAs as indicated. Proteins were detected using the antibodies indicated except MLL-AF4, which was detected with an AF4-C antibody. (D) A summary of AF4 protein interactions. (E) MLL-N, AF4-C, AFF4, ENL, AF9, and Cyclin T1 ChIP + real-time PCR with scrambled control versus MLL-AF4 siRNA-treated SEM cells from (A). Values and error bars represent the mean ± SD of at least two independent ChIP experiments. Primer sets are as in Figure 1B, 1C, and 2A.

Guenther et al. (2008) previously rejected *RUNX1* as a potential MLL-AF4 target gene because it displayed MLL-N, MLL-C, AF4-C binding, and H3K4Me3 and H3K79Me2 in both SEM and the control REH (non-MLLr) cell lines. To determine if MLL-AF4 is a key regulator of *RUNX1* expression, MLL-AF4-specific siRNA (Thomas et al., 2005) knockdowns were performed in RS4;11 and SEM cell lines. At both the RNA and protein levels, we saw an MLL-AF4-dependent loss of RUNX1 expression (Figures 3A–3C). Importantly, we also found that wild-type MLL had no effect on HOXA9 or RUNX1 regulation (Figure 3B), suggesting that MLL-AF4, but not wild-type MLL, is key to maintaining the expression of these target genes.

Because MLL-AF4 is key to maintaining *HOXA9* and *RUNX1* target gene expression, we wanted to determine if MLL-AF4 was responsible for assembling a specific complex at these target genes in vivo. The AF4-C portion of MLL-AF4 interacts directly with wild-type AF9, ENL, and AFF4, and weakly homodimerizes with wild-type AF4, providing an indirect interaction between MLL-AF4 and the Cyclin T1/CDK9 pTEFb complex (Benedikt et al., 2011; Biswas et al., 2011; Lin et al., 2010; Mueller et al., 2007; Yokoyama et al., 2010) (Figure 3D). In SEM cells, specific siRNA knockdowns of MLL-AF4 reduced binding of MLL-N, AF4-C, and the MLL-AF4 interacting factors ENL and AF9 at *RUNX1, HOXA9, HOXA10,* and *CDKN1B* (Figure 3E), but had no effect on the binding of Cyclin T1 and AFF4. Together, these results indicate that *RUNX1, HOXA9, HOXA10,* and *CDKN1B* are direct targets of MLL-AF4, and that MLL-AF4 stabilizes the recruitment of AF9 and ENL, but not Cyclin T1 or AFF4.

### t(4;11) Cell Lines Support Higher Levels of *RUNX1* Expression Than Other MLL-FP Leukemias

**Figure 4:**
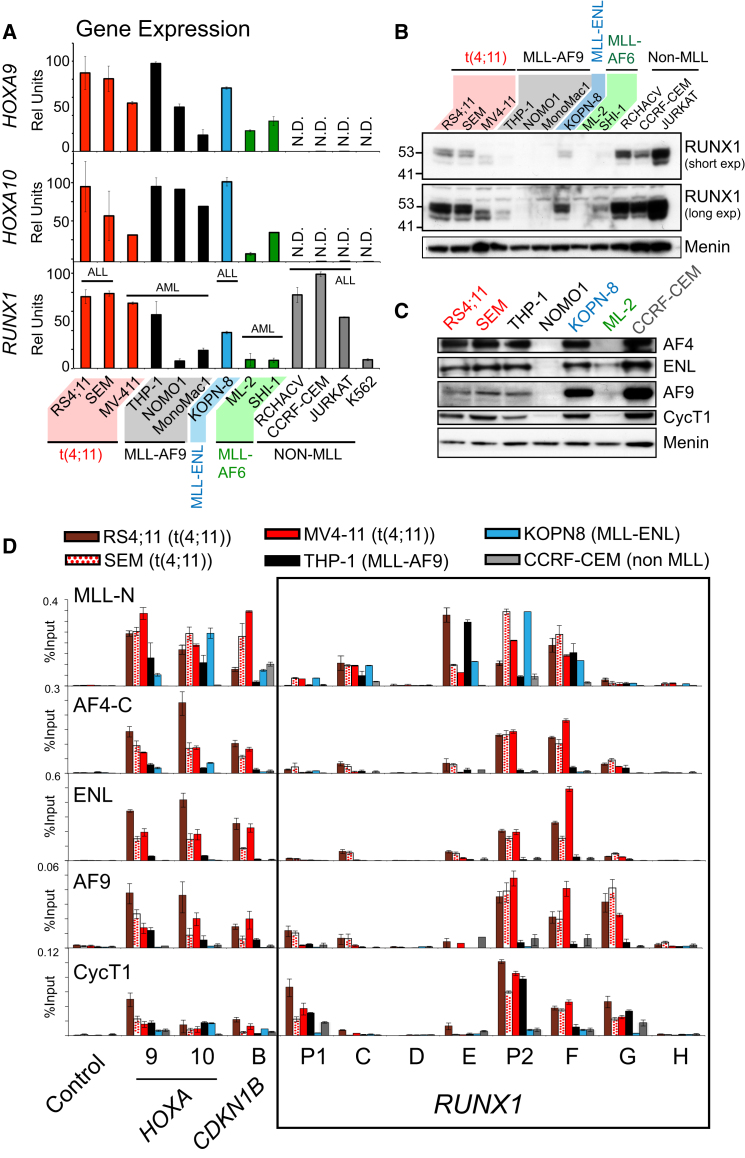
In t(4;11) Cells, *RUNX1* Is Highly Expressed and Has High Levels of ENL and AF9 Bound to the Locus (A) Real-time PCR quantification (see gene expression analysis in Extended Experimental Procedures) of *HOXA9* (top), *HOXA10* (middle), and *RUNX1* (bottom) gene expression in patient cell lines. The cell lines analyzed are: RS4;11 (t-4;11), SEM (t-4;11), MV4-11 (t-4;11), THP-1 (MLL-AF9), NOMO-1 (MLL-AF9), MONO-MAC1 (MLL-AF9), KOPN-8 (MLL-ENL), ML-2 (MLL-AF6 and an MLL deletion), SHI-1 (MLL-AF6), RCH-ACV (normal MLL), CCRF-CEM (normal MLL), JURKAT (normal MLL), and K562 (normal MLL). Error bars represent the ±SD of two independent experiments. ALL, acute lymphoblastic leukemia; AML, acute myeloid leukemia; N.D., not detected. (B) Western blot of RUNX1 in the cell lines as described in (A) with a short exposure (top panel) and a long exposure (middle panel). (C) Western blot of nuclear extracts in the cell lines indicated and probed with the antibodies as indicated. (D) MLL-N, AF4-C, ENL, AF9, and Cyclin T1 ChIP in RS4;11 (dark red bars), SEM (spotted red bars), MV4-11 (bright red bars), THP-1 (black bars), KOPN-8 (blue bars), and CCRF-CEM (gray bars) patient cell lines. The control primer set is from a random gene-poor region on human chromosome 8; otherwise, primer sets are as indicated in Figures 1B, 1C, and 2A. Error bars represent the ±SD of two independent experiments. See also Figure S3.

To further analyze the potential importance of *RUNX1* in t(4;11) leukemias, we compared gene expression patterns and complex assembly at target genes in different MLL-FP cell lines. Typically, both *HOXA9* and *HOXA10* are highly expressed in MLL-FP cell lines and show almost no expression in non-MLL-FP cell lines (Figure 4A, top and middle). Although *RUNX1* gene expression is complicated by the fact that it appears to be generally higher in ALLs compared to AMLs, consistent with the primary patient data in Figure 2H, *RUNX1* expression is upregulated in t(4;11)-containing cells compared to other MLL-FPs (Figure 4A, bottom). In general, although there are some unique isoforms specific to different cell types (perhaps reflecting myeloid versus lymphoid origins), RUNX1 protein levels are higher in t(4;11) cells than in other MLL-FP cells (Figure 4B).

**Figure S3:**
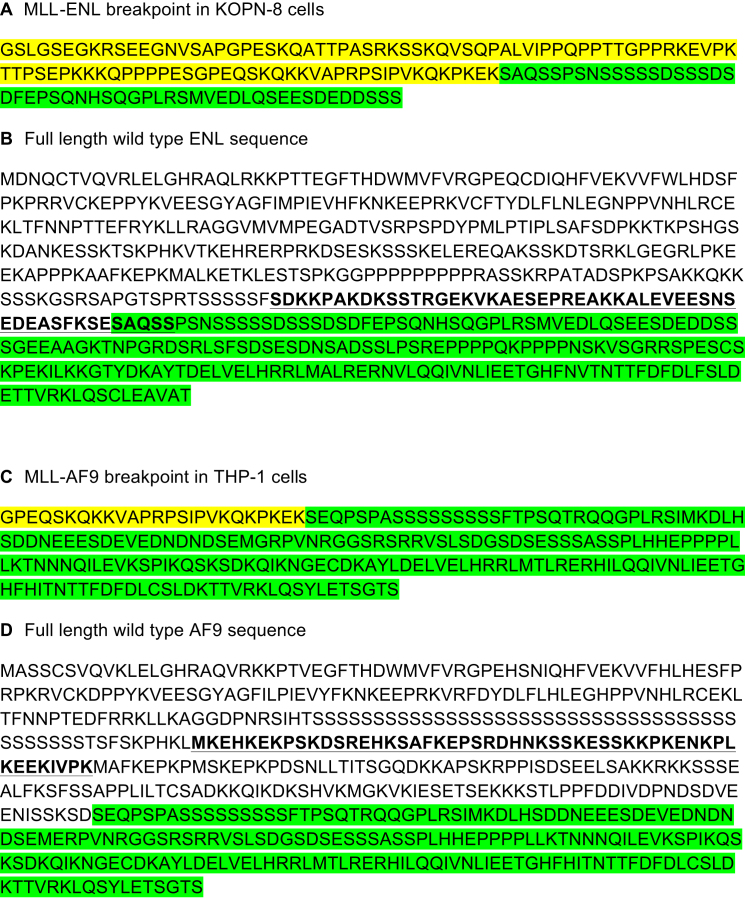
Antibody Epitopes Compared to MLL-FP Sequences in KOPN-8 (MLLENL) and THP-1 (MLL-AF9) Cells, Related to Figure 4 (A) Fusion sequence between MLL ex8 (yellow) and ENL ex7 (green) in KOPN-8 cells. The breakpoint was determined by sequencing cDNA from KOPN-8 cells. (B) Wild-type ENL protein sequence, sequence in KOPN-8 MLL-ENL fusion (green) and sequence recognized by Bethyl antibody A302-268A (Bold, underline). (C) Fusion sequence between MLL ex8 (yellow) and AF9 ex5 (green) in THP-1 cells, sequence taken from Odero et al. (2000). (D) Wild-type AF9 protein sequence, sequence in THP-1 MLL-AF9 fusion (green) and sequence recognized by Bethyl antibody A300-595A (Bold, underline).

Interestingly, although ENL, AF9, and Cyclin T1 are all expressed in several cell lines that all express RUNX1 (Figure 4C), wild-type AF9 and ENL (see Figure S3) binding is more highly enriched at *RUNX1* and other target genes in t(4;11) cells than in the other cell lines (Figure 4D). Although MLL-N ChIP cannot specifically detect MLL-AF9 or MLL-ENL (Figure 4D), in combination with the data in Figure 3, we think the most likely explanation for these results is that MLL-AF4 differs from other MLL-FPs and increases stable AF9 and ENL binding at *RUNX1*.

### RUNX1 Is Required for the Growth of t(4;11) Cells

**Figure 5:**
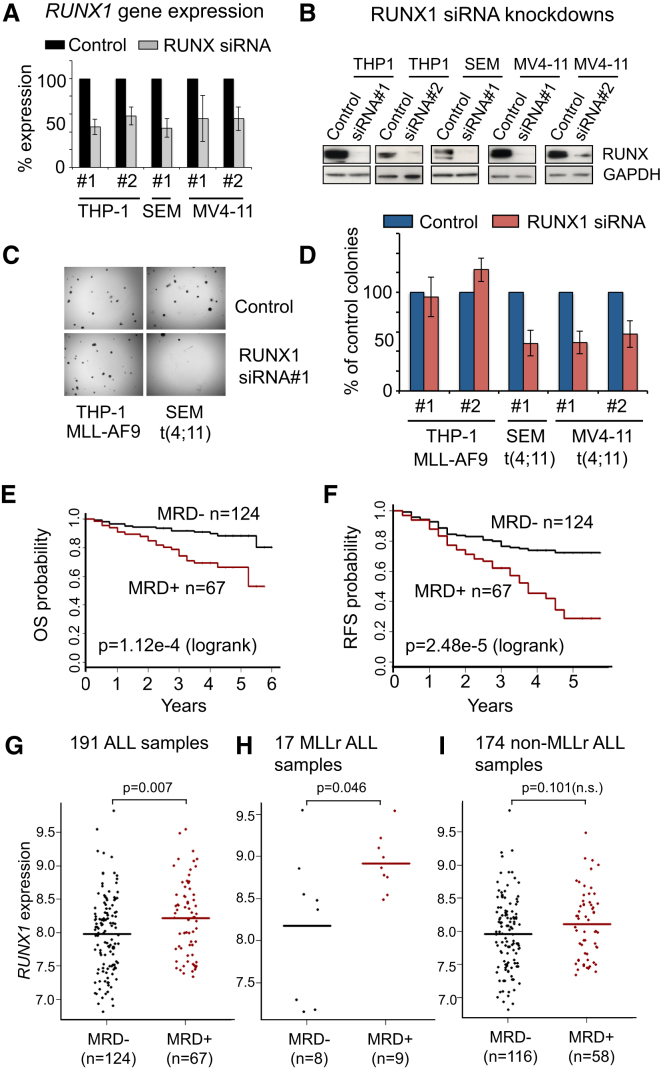
High-Level *RUNX1* Expression Is Important for t(4;11) Cell Growth and Correlates with a Poor Clinical Prognosis in *MLL*-Rearranged Leukemias (A) Real-time PCR expression of *RUNX1* in THP-1 (MLL-AF9), SEM (t-4;11), and MV4-11 (t-4;11) cells treated with either a nontargeting control siRNA or two different *RUNX1* siRNAs (#1 and #2). Data for THP-1#1 and SEM#1 are the mean ± SD of six independent experiments. The rest of the data are the mean ± SD of three independent experiments. Samples for gene expression analysis were taken the day of colony assay plating. (B) Representative western blots from samples in (A) probed with either RUNX1 or GAPDH antibodies. (C) Representative photomicrographs of THP-1 (left column) and SEM (right column) clonogenic cultures after treatment with either a nontargeting control (top row) or with *RUNX1* siRNA#1 (bottom row). (D) Colony counts 14 days after plating. Data are the mean ± SD of either six independent experiments (THP-1#1 and SEM#1) or three independent experiments (the rest). Three replicates were plated per experiment. Control samples were set at 100% for each individual experiment. (E and F) Kaplan-Meier estimates of overall survival (OS) and relapse-free survival (RFS) based on minimal residual disease (MRD) measured at day 29 of the end-induction among 191 COG P9906 (Harvey et al., 2010) ALL patients, log rank test p values. (G) A total of 67 MRD+ patients had higher average *RUNX1* expression levels than 124 MRD− patients (p = 0.00746). (H) Among 17 MLLr patients, 9 patients that were MRD+ had significantly higher levels of *RUNX1* expression than 8 MRD- patients (p = 0.0464, two-tailed Wilcoxcon test). (I) Among 174 non-MLLr B-ALL patients, 58 patients who were MRD+ had no significant increase in *RUNX1* expression (p = 0.101, two-tailed Wilcoxon test). See also Figure S4.

**Figure S4:**
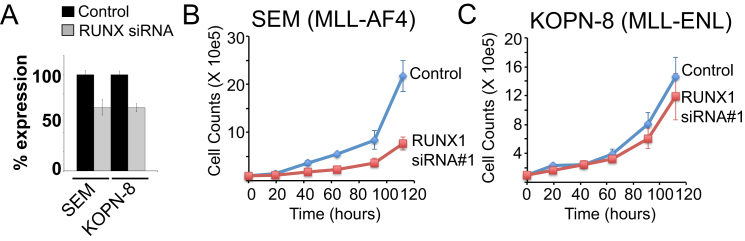
*RUNX1* Expression Is Important for t(4;11) But Not MLL-ENL Growth, Related to Figure 5 (A) Real Time PCR expression of *RUNX1* in SEM (t-4;11) or KOPN-8 (MLL-ENL) cells treated with either a non-targeting control siRNA or a *RUNX1* siRNA (#1). Error bars represent the ± SD of three separate PCR reactions. (B and C) Cell counts of the control (blue line) or RUNX1 siRNA (red line) treated cells from (A) in SEM (B) or KOPN-8 (C) cells over ∼5 days. Error bars represent the ± SD of two independent experiments.

To determine if *RUNX1* expression is important for the leukemic growth of different MLL-FPs, we used colony-forming assays coupled with *RUNX1* siRNA knockdowns in SEM (t-4;11), MV4-11 (t-4;11), and THP-1 (MLL-AF9) cells. Cells collected 24 hr after plating contained ∼50% of *RUNX1* mRNA compared to a nontargeting siRNA control (Figure 5A) and resulted in a large reduction in RUNX1 protein levels (Figure 5B). In SEM and MV4-11 cells, *RUNX1* siRNA treatment inhibited clonogenic ability by ∼60% after 14 days, while little effect was observed in THP-1 cells (Figures 5C and 5D). Similar t(4;11) sensitivity to *RUNX1* levels was observed in cell growth assays comparing SEM cells and KOPN-8 cells after *RUNX1* siRNA treatment (Figures S4A–S4C). Together, these results suggest that *RUNX1* expression specifically contributes to the growth of t(4;11) cells but not other common MLL-FPs.

### High *RUNX1* Expression Correlates with a Poor Clinical Outcome in ALL

Minimal residual disease (MRD) after treatment is generally considered to be an indicator of poor prognosis. In the COG P9906 clinical trial (Harvey et al., 2010), 191 out of 207 ALL patients had MRD data available. As expected, the 67 MRD+ patients had a significantly worse overall survival and relapse-free survival than the 124 MRD− cases (Figures 5E and 5F). We found that the 124 MRD− patients had a significantly lower average level of *RUNX1* expression compared to the 67 MRD+ patients (Figure 5G). Among the 191 patients, 17 harbor MLL-FPs (MLLr), among which, the 9 patients that were MRD+ at day 29 had a higher average *RUNX1* expression than the 8 patients that did not (Figure 5H). Interestingly, when these MLLr patients are removed from the data set, the resulting 174 non-MLLr ALL patients showed no significant correlation between *RUNX1* expression and MRD status (Figure 5I). Although we unfortunately do not have specific data for t(4;11) leukemias, the correlation between higher *RUNX1* expression levels and worse clinical outcomes in MLLr patients suggests that *RUNX1* expression can directly contribute to leukemogenesis in human patients.

### RUNX1 Activates Target Genes in t(4;11) Leukemic Cells

**Figure 6:**
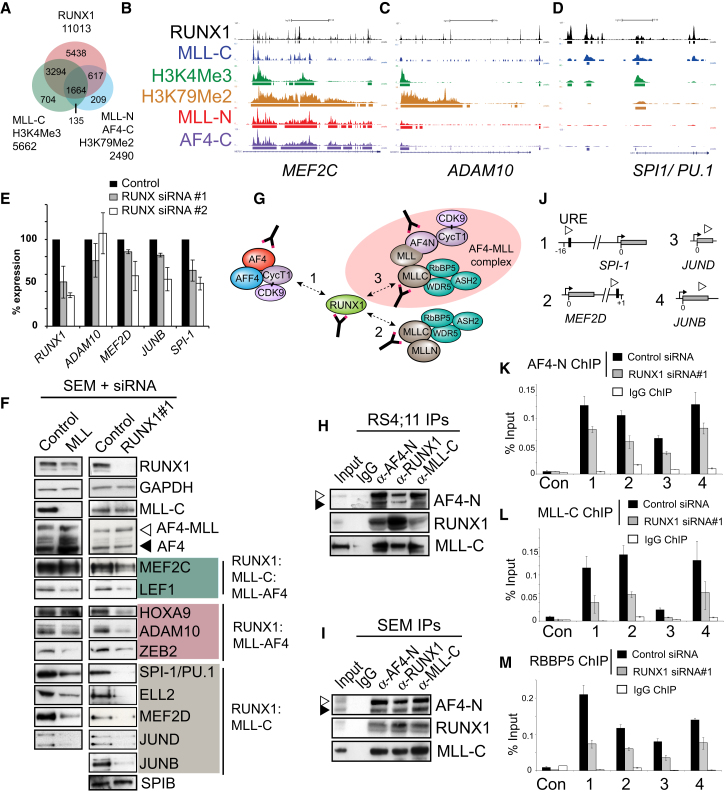
RUNX1 Interacts with the AF4-MLL Complex and Activates Gene Targets (A) RUNX1 ChIP-seq in SEM cells compared with MLL-C:H3K4Me3 and MLL-N:AF4-C:H3K79Me2 ChIP-seq. (B–D) Sample ChIP-seq tracks from SEM cells across *MEF2C* (B), *ADAM10* (C), and *SPI1/PU.1* (D). (E) Gene expression analysis by real-time PCR in SEM cells treated with two different *RUNX1* siRNAs (gray bars, siRNA#1; white bars, siRNA#2). For each experiment, the PCR signal was quantified relative to control-treated cells. Results represent the mean ± SD of three independent knockdown experiments. (F) Western blots as indicated in SEM cells treated with a nontargeting control, *RUNX1* siRNA#1, or a wild-type MLL siRNA. (G) RUNX1 protein complex interactions. RUNX1 can interact with a wild-type AF4 complex (interaction 1), a wild-type MLL complex (interaction 2), and potentially with an AF4-MLL complex (interaction 3). (H and I) Immunoprecipitation (IP) experiments using RS4;11 (H) and SEM (I) nuclear extracts. Extracts were IP’d with αIgG (lane 2), αAF4-N (lane 3), αRUNX1 (lane 4) or αMLL-C (lane 5), blotted and probed with the antibodies indicated. Input lanes represent 1% of the amount of extract used for the IPs. (J) A schematic of the *MEF2D*, *JUNB*, *JUND,* and *SPI-1* (aka *PU.1*) loci showing the approximate location of PCR primer sets (open arrow heads) used for ChIP analysis. Black box indicates consensus RUNX1 binding motifs in the upstream regulatory region (URE) of *SPI-1* (Huang et al., 2008; Huang et al., 2011) and the first intron of *MEF2D* (Pencovich et al., 2011). Gray box indicates exon 1 of *MEF2D*, *JUND*, *JUNB,* and *SPI-1*. (K–M) ChIP analysis in SEM cells treated with a nontargeting control or *RUNX1* siRNA#1 at the targets as indicated using antibodies to AF4-N (K), MLL-C (L), and RBBP5 (M). Error bars represent the ±SD of three separate PCR reactions. See also Table S5 and Figure S5.

**Figure S5:**
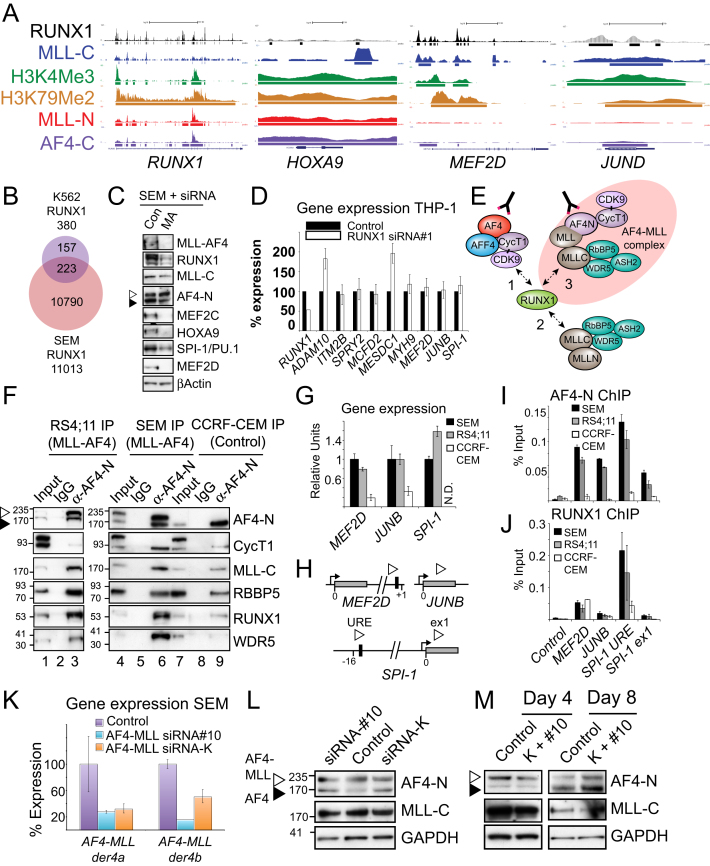
RUNX1 ChIPseq and AF4-MLL Complex Data, Related to Figure 6 (A) Sample ChIP-seq tracks from SEM cells across *RUNX1*, *HOXA9*, *MEF2D* and *JUND*. (B) ChIP-seq overlap between the RUNX1 SEM cell target gene set versus the set of RUNX1 target genes from (Pencovich et al., 2011). (C) Western blots for the proteins indicated in SEM cells treated with a scrambled control or an MLL-AF4 siRNA. Proteins were detected using the antibodies indicated except MLL-AF4, which was detected with an AF4-C antibody. (D) Gene expression analysis of selected genes in THP-1 cells treated with *RUNX1* siRNA (siRNA#1). For each experiment, the PCR signal was quantified relative to the appropriate control treated cells. Results represent the average of three independent knockdown experiments, and error bars represent the standard deviation between experiments. (E) A schematic of protein complex interactions centering on the RUNX1 protein. RUNX1 can interact with a wild-type AF4 complex (interaction 1) through CyclinT1 (Elagib et al., 2008; Jiang et al., 2005), a wild-type MLL-C complex (interaction 2) and potentially with an AF4-MLL complex (interaction 3). (F) Immunoprecipitation (IP) experiments using RS4;11 (t(4;11)), SEM (t(4;11)) and CCRF-CEM (wild-type MLL1) nuclear extracts. Extracts were IP’d with either αAF4-N (lane 3, 6 and 9) or a control αIgG (lane 2, 5 and 8) antibody, blotted and probed with the antibodies indicated. Lane 1,4 and 7 (Inputs) represents 1% of the amount of extract used for the IPs. AF4-MLL is indicated by a white arrowhead (AF4-MLL is 328 KDa, the white arrowhead represents the ∼194KDa Taspase 1 cleaved product) while wild-type AF4 is indicated by a black arrowhead (wild-type AF4 has a predicted size of 131 KDa but an apparent MW of 175 KDa). MLL-C is the Taspase 1 cleaved product of both AF4-MLL and wild-type MLL which is 134KDa but runs with an apparent MW of 180KDa. (G) Real Time PCR of *MEF2D*, *JUNB* and *SPI-1* expression in SEM, RS4;11 and CCRF-CEM cells. (H) A schematic of the *MEF2D*, *JUNB* and *SPI-1* (aka *PU.1*) loci showing the approximate location of PCR primer sets (open arrow heads) used for ChIP analysis. Black box = consensus RUNX1 binding motifs in the upstream regulatory region (URE) of *SPI-1* (Huang et al., 2008; Huang et al., 2011). and the first intron of *MEF2D* (Pencovich et al., 2011). Grey box = exon1 of *MEF2D*, *JUNB* and *SPI-1*. (I and J) ChIP analysis in SEM (black bars), RS4;11 (gray bars), CCRF-CEM (white bars) at the targets as indicated using antibodies to AF4-N (I) or RUNX1 (J). (K) Real Time PCR expression of *AF4-MLLder4a* and *der4b* (Kumar et al., 2011) in SEM cells treated with a scrambled control (purple bars), an AF4-MLL siRNA (siRNA#10, blue bars) or an AF4-MLL siRNA (siRNA-K, orange bars) from (Kumar et al., 2011). Error bars represent the ± SD of three separate PCR reactions. In each individual experiment, control values were arbitrarily set to 100. AF4-MLL siRNA sequences are in supplemental methods. (L) Western blots of the AF4-MLL knockdowns in (K) using the antibodies indicated. The apparent MW of AF4-MLL and MLL-C is explained in (F) above. (M) Western blots at day 4 and day 8 of SEM cells treated with both AF4-MLL siRNA-K and siRNA#10 at day 0, day 2, day 4 and day 7. Antibodies are as indicated. AF4-MLL is indicated by a white arrowhead while wild-type AF4 is indicated by a black arrowhead as explained in (F) above.

To understand the function of the RUNX1 protein in t(4;11) leukemic cells, we performed RUNX1 ChIP-seq in SEM cells and identified 11,013 genes directly bound by the RUNX1 protein (Figures 6A–6D and S5A; Table S5). Interestingly, recent work has shown that RUNX1 can interact with the wild-type MLL protein complex (Huang et al., 2011), and we found 3,294 genes that show a specific overlap between MLL-C/H3K4Me3 binding and RUNX1 (Figures 6A–6D and S5A; Table S5). RUNX1 also binds to 617 MLL-AF4 targets (i.e., MLL-N/AF4-C/H3K79Me2 binding sites, Figures 6A and 6C) and 1,664 genes where all the proteins overlap (Figure 6A; Table S5), including *MEF2C* (Figure 6B) and the *RUNX1* gene itself (Figure S5A).

*MEF2D* and *JUNB* were both previously identified among a set of 380 genes tightly regulated by RUNX1 in K562 cells (Pencovich et al., 2011), whereas *SPI-1* (aka *PU.1*) is a previously identified important target gene in RUNX1-mediated leukemogenesis (Huang et al., 2008, 2011). *MEF2D*, *JUNB,* and *SPI-1* are all bound by RUNX1 in SEM cells (Figure 6A, S5A, and S5B; Table S5). Interestingly, loss of RUNX1 protein levels appears to have the strongest effect on target genes bound primarily by RUNX1 and MLL-C (Figures 6E and 6F).

Wild-type MLL knockdowns reduce expression of some MLL-C/RUNX1-bound gene targets, although not to the same degree as knockdowns of RUNX1 (Figure 6F). Even though MLL-AF4 does not bind directly to *SPI-1* or *MEF2D* (Figures 6D and S5A), MLL-AF4-specific knockdowns reduce expression of both of these target genes, likely due to the reduction of RUNX1 protein levels (Figure S5C). Importantly, RUNX1 knockdowns in THP-1 cells did not reduce target gene expression, and in some cases actually increased expression of RUNX1 target genes (Figure S5D). Taken as a whole, these data suggest that RUNX1 is functioning as an activator at certain key target genes in t(4;11) SEM cells, and MLL-C:RUNX1-bound target genes are particularly sensitive to the loss of RUNX1.

### RUNX1 Activates Gene Targets in t(4;11) Cells by Cooperating with an AF4-MLL Complex

Past work revealed that AF4-MLL is expressed in human patients (Kowarz et al., 2007) and contributes to t(4;11) leukemogenesis (Bursen et al., 2004, 2010). AF4-MLL can alter the epigenetic profile of target genes by interacting with components of the SEC and the wild-type MLL-C complex (Benedikt et al., 2011; Figure 6G), but AF4-MLL does not function primarily through the activation of canonical MLL-AF4 target genes such as *HOXA9* (Bursen et al., 2010). RUNX1 directly interacts with the C-terminal SET domain of MLL (Huang et al., 2011), suggesting that RUNX1 could be a component of a wild-type MLL and an AF4-MLL:MLL-C complex (Figure 6G, interactions 2 and 3, respectively).

To determine if RUNX1 exists in a complex with AF4-MLL (see Figure 6G), we performed immunoprecipitation (IP) experiments with RS4;11 and SEM nuclear extracts (Figures 6H and 6I). We found that αRUNX1, αMLL-C, and αAF4-N could coIP a complex containing RUNX1, MLL-C, wild-type AF4 (black arrowhead), and a band that corresponds to the cleaved ∼194 kDa AF4-MLL protein (white arrowhead; see the legend for Figure S5F for an explanation of the apparent molecular weights of these proteins). AF4-N IPs in CCRF-CEM nuclear extracts failed to detect this 194 KDa AF4-MLL band, and were less enriched for RUNX1 and the MLL-C complex than comparable IPs in SEM or RS4;11 cells (Figures S5E and S5F). Together, these results support the possibility that AF4-MLL exists in a complex with both MLL-C and RUNX1.

**Figure 7:**
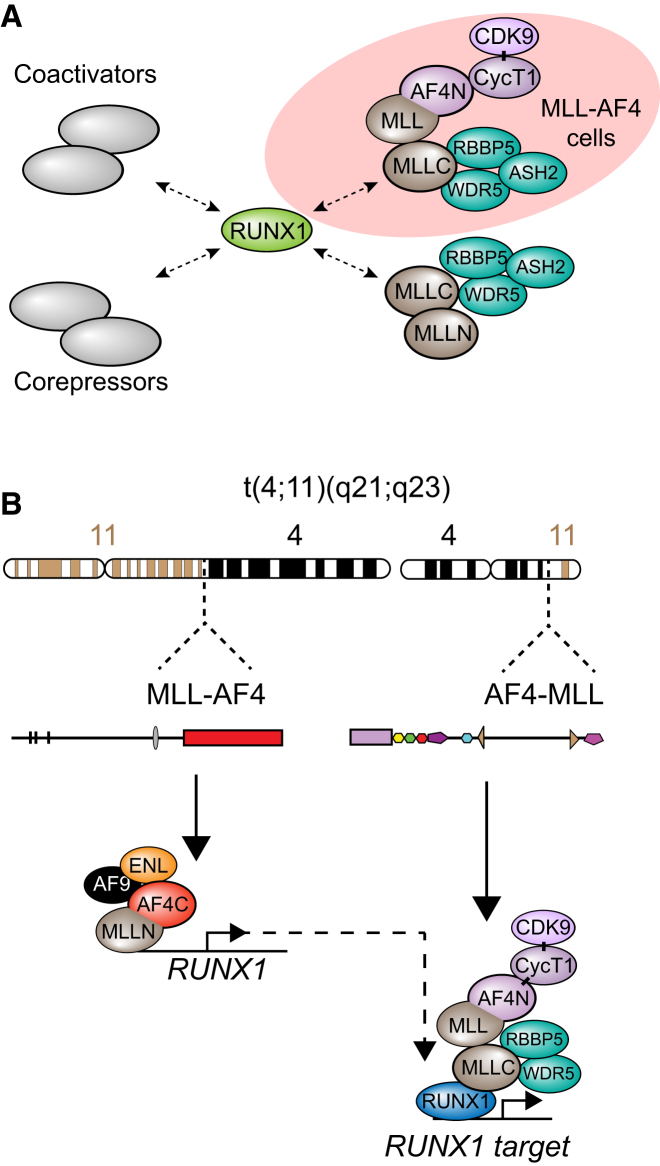
MLL-AF4 Activates the *RUNX1* Gene and the RUNX1 Protein Interacts with the AF4-MLL Complex and Activates Gene Targets (A) RUNX1 can interact with either coactivators or corepressors to cause gene activation or repression. In t(4;11) cells, RUNX1 can also interact with the AF4-MLL complex. (B) In t(4;11) leukemias, *MLL-AF4* is expressed from one translocated chromosome, and the MLL-AF4 protein binds to and activates the *RUNX1* gene by stabilizing AF9 and ENL binding. *AF4-MLL* is expressed from the other translocated chromosome, and the RUNX1 protein interacts with the AF4-MLL complex and binds to target genes.

RUNX1 siRNA experiments that reduce expression of SPI-1, MEF2D, JUND, and JUNB (Figure 6F) disrupt binding of AF4-N, MLL-C, and the MLL-C complex component RBBP5 to these target genes in vivo (Figures 6J–6M). Further, expression of *MEF2D*, *JUNB,* and *SPI-1* is higher in SEM and RS4;11 cells than in CCRF-CEM cells (Figure S5G), and this correlates with an increased binding of AF4-N (Figures S5H and S5I). Increased AF4-N binding is seen even at the *MEF2D* target gene, which has approximately equal levels of RUNX1 binding in CCRF-CEM cells compared to RS4;11 and SEM cells (Figures S5I and S5J). Unfortunately, AF4-MLL-specific siRNAs failed to reduce AF4-MLL protein levels (Figures S5K–S5M), and we were not able to directly test whether AF4-MLL regulates RUNX1 target genes. However, taken as whole, these data show that RUNX1 activates certain key target genes in t(4;11) pre-B-ALL cells, and it might accomplish this through recruitment of an RUNX1:MLL-C:AF4-MLL complex (Figure 7).

## Discussion

MLL-FPs are thought to promote leukemogenesis through the epigenetic activation and maintenance of master regulatory factors such as *HOXA9* and *MEIS1,* which set up gene expression networks responsible for constitutive activation of cellular growth and proliferation pathways. However, in t(4;11) patient samples, half of the leukemias analyzed do not have elevated levels of *HOXA* expression (Stam et al., 2010; Trentin et al., 2009), and low-level *HOXA* expression actually correlates with a worse prognosis (Stam et al., 2010). Furthermore, AF4-MLL is able to induce leukemias in mice without activating *HOXA* or *MEIS1* expression (Bursen et al., 2010). Together, these results suggest that t(4;11) leukemias may activate alternate pathways that are not dependent on *HOXA* or *MEIS1* expression.

In this analysis, we have identified a 491 target gene set that is generally highly expressed among MLLr leukemias. *RUNX1* is a unique exception to this in that it is specifically overexpressed in t(4;11) leukemias (Figure 2H and Montero-Ruíz et al., 2012). RUNX1 siRNA knockdowns inhibited clonogenicity of t(4;11) (SEM and MV4-11) cells but not MLL-AF9 (THP-1) cells, indicating that the oncogenic role for RUNX1 in t(4;11) leukemia appears to be t(4;11) specific but lineage independent, with both B-ALL (SEM) and AML (MV4-11) affected.

RUNX1 is known to play critical roles in hematopoiesis (Mangan and Speck, 2011), and is commonly mutated in leukemia as a tumor suppressor (Blyth et al., 2005; Mangan and Speck, 2011; Zhang et al., 2012), including in MLL-ENL driven leukemias (Nishimoto et al., 2011). However, RUNX1 overexpression in childhood leukemias has been reported (Mikhail et al., 2002; Niini et al., 2002), whereas oncogenic function has been identified in other cancers, such as T cell lymphomas (Blyth et al., 2005).

Recent analyses have suggested that MLL-AF4 promotes transcription elongation by stabilizing the binding of factors such as pTEFb, DOT1L, ELL, AFF4, AF9, and ENL at target genes in vivo (Lin et al., 2010; Yokoyama et al., 2010). AF4-MLL has been shown to activate gene targets through a similar ability to promote transcription elongation by interacting with a pTEFb-containing complex (Benedikt et al., 2011). Here, we show that the RUNX1 protein can interact with an AF4-MLL complex and stabilize its binding to certain gene targets. Thus, leukemic cells that express AF4-MLL produce an additional coactivator complex (Benedikt et al., 2011) that may push the balance toward RUNX1 functioning as a general activator, and this may have an impact on whether *RUNX1* is a tumor suppressor or an oncogene in different cell types (Figure 7A).

Kumar et al. (2011) reported that an AF4-MLL-specific siRNA had no effect on the growth of the SEM t(4;11) leukemia cell line. However, as was pointed out in a rebuttal article (Marschalek, 2011), the specific AF4-MLL siRNA used was not likely to produce a knockdown of the AF4-MLL protein, something we have now confirmed in our results here (Figures S5K–S5M). Unfortunately, our own attempt to design an AF4-MLL-specific siRNA was also unsuccessful (Figures S5K–S5M), likely due to the stability and low turnover of the AF4-MLL protein (Marschalek, 2011), so the specific role of AF4-MLL remains to be definitively elucidated.

The data we present are consistent with an interplay between MLL-AF4 and AF4-MLL through the regulation and function of RUNX1, providing a model of how these oncoproteins could cooperate on a molecular level (Figure 7B). Such a cooperative effect between these two fusion proteins could explain why this particular *MLL* translocation produces such an aggressive leukemia with relatively few additional mutations (Bardini et al., 2011; Bardini et al., 2010).

## Experimental Procedures

### Chromatin Immunoprecipitation Assays

ChIP (for both real-time PCR and ChIP-seq) experiments were performed as described in Milne et al. (2009), with several modifications, as outlined in Extended Experimental Procedures.

### ChIP-Seq Analysis

The RS4;11 MLL-N (Akalin et al., 2012) and RS4;11 MLL-N, AF4-C, and H3K79Me2 (Geng et al., 2012) ChIP-seq data have also been used in separate studies analyzing DNA hypomethylation at target genes. The SEM MLL-N, AF4-C, and H3K79Me2 is from Guenther et al. (2008). Regions of overlap for MLL-N and AF4-C were defined as peaks overlapping in the promoter regions (± 2 kb to transcriptional start site [TSS]), and for H3K79Me2 as the gene body regions (−2 kb to TSS to +1 kb to transcriptional end SITE [TES]). Further details of analysis are included in Extended Experimental Procedures.

### Patient Data

Gene expression microarray data from three large cohorts of patients with ALL were analyzed, including the ECOG Clinical Trial E2993, (Geng et al., 2012), the COG Clinical Trial P9906 (Harvey et al., 2010), and the St. Jude Research Hospital pediatric ALL clinical trial (Ross et al., 2003). Further details are provided in Extended Experimental Procedures.

### Colony Forming Assays

Twenty-four hours after second transfection, cells were plated at a density of 1, 2, or 2.5 × 10^5^ cells/ml, in triplicate, plated in IMDM MethoCult media (H4100; STEMCELL Technologies) supplemented with fetal calf serum and cultured for 14 days (37°C, 5% CO_2_) before counting. Colony-forming assays were run in triplicate with at least three biological repeats.

### Western Blotting

A total of 10 μg nuclear extract was loaded per lane on NuPAGE 4%–12% BisTris gels (Life Technologies) and blotted onto polyvinylidene fluoride membrane (Immobilon) at 100V for 1 hr using a Tris-glycine blotting buffer. Blots were probed with the antibodies indicated.

Extended Experimental Procedures **Cell Culture and Cell Lines**

SEM (Greil et al., 1994) and SHI-1 (Chen et al., 2005) cells (from DSMZ, http://www.cell-lines.de) were cultured in IMDM (GIBCO) supplemented with 15% FCS. RS4;11, MV4-11, THP-1, CCRF-CEM (from ATCC http://www.lgcstandards-atcc.org) were cultured in RPMI (GIBCO) supplemented with 15% FCS. NOMO1 (Kato et al., 1986), MonoMAC1 (Ziegler-Heitbrock et al., 1988), KOPN-8 (Matsuo and Drexler, 1998), ML-2 (Ohyashiki et al., 1986), RCH-ACV (Jack et al., 1986) cells (from DSMZ, http://www.cell-lines.de), Jurkat and K562 cells were cultured in RPMI (GIBCO) supplemented with 15% FCS.

**Colony and Cell Growth Assays**

24 hr post second transfection cells were plated at a density of 1, 2 or 2.5 × 10^5^ cells per ml, in triplicate. Cells used for colony forming assays were transferred into IMDM (GIBCO) supplemented with 20% FCS and passaged twice before transfection and plating. 4 × 10^3^ MV4-11 cells were plated in 30% FBS with 10^−4^ M (0.1mM) 2-mercaptoetanol supplemented IMDM Methocult media for the colony assay. For the cell growth assay, manual viable cell counts were performed using 0.4% Trypan blue (GIBCO, Life technologies) and a Neubauer haemocytometer, at the times indicated.

**Sequencing of MLL-FP Breakpoints**

The THP-1 cell breakpoint in Figure S3 was taken from (Odero et al., 2000). The MLL-ENL breakpoint in KOPN-8 cells was determined by sequencing a PCR fragment from KOPN-8 cDNA using the following primers: MLLex5for: GAGGATCCTGCCCCAAAGAAAAG, ENLrev: GACGAAGAGTCGTCCTCGTCGGACT.

**Chromatin Immunoprecipitation Assays**

ChIP (for both Real Time PCR and ChIP-seq) experiments were performed as described in (Milne et al., 2009) with the following modifications: H3K79Me2 and H3K4Me3 ChIP samples were fixed using a 1% formaldehyde (FA) fixation protocol for 10 min, while a 45 min, 2mM DSG and a 30 min 1% FA double fixation protocol was used for all other antibodies. Fixed chromatin samples were fragmented using a Bioruptor sonicator (Diagenode) for 30 min at high in a constantly circulating 4°C water bath to an average size of 200-500bps. AF4-N ChIP signal was improved by reducing the sonication time from 30 min to 20 min. Antibody:chromatin complexes were collected with a mixture of protein A and Protein G Dynabeads (Life Technologies) collected with a magnet, and washed 2X with a solution of 50mM HEPES-KOH, pH 7.6, 500mM LiCl, 1mM EDTA, 1% NP-40, 0.7% Na-Deoxycholate. After a TE wash, samples were eluted, RNase and Proteinase K treated, and purified using a QIAGEN PCR purification kit. ChIP samples were quantified relative to inputs as described in (Milne et al., 2009). Briefly, the amount of genomic DNA coprecipitated with antibody is calculated as a percentage of total input using the following formula Δ*C*_*T*_ = *C*_*T*_ (input) – *C*_*T*_ (chromatin IP), % total = 2^**Δ**CT^ X 5.0%. A 50 μl aliquot taken from each of 1 ml of sonicated, diluted chromatin before antibody incubation serves as the input, thus the signal from the input samples represents 5% of the total chromatin used in each ChIP. CT values are determined by choosing threshold values in the linear range of each PCR reaction.

**Primers for ChIP**

SYBR green primer sets were used for all ChIP figures. ChIP signal was calculated as a % of input as described above. Control region, For: GGCTCCTGTAACCAACCACTACC, Rev: CCTCTGGGCTGGCTTCATTC; HOXA9, For: ATGCTTGTGGTTCTCCTCCAGTTG, Rev: CCGCCGCTCTCATTCTCAGC; HOXA10, For: CGCAACCACCCCAGCCAG, Rev: TTGTCCGCCGAGTCGTAGAGG; CDKN1B-A, For: TCTTCTTCGTCAGCCTCCCTTC, Rev: TCGCAGAGCCGTGAGCAAGC; CDKN1B-B, For: TGCCGTAACAGGGTGATTTGG, Rev: CTCCACTTCCTTTGTGCTGGG; RUNX1-P1, For: GAACCACAAGTTGGGTAGCCTGG, Rev: GATTCGTCCTGCCTGCTGACC; RUNX1-C, For: CAACTGTGAGCCGAAAGGGAAGAC, Rev: GAAGGGAACAATGGTTTGCTTGG; RUNX1-D, For: AGATTCTCTTCGGCTTTCCCACTC, Rev: GCTGGCATTTGAACACAGGCTC; RUNX1-E, For: TGCGAGAGCGAGAAAACCACAG, Rev: GCAGAAAGCAACAGCCAGAAACG; RUNX1-P2, For: GACGCACGCAGCAAGTGAGAC, Rev: TGGGTCGGTTTCTGTAATGGGTG; RUNX1-F, For: CCCTGTCGCCGTCTGGTAGG, Rev: AACGCCTCGCTCATCTTGCC; RUNX1-G, For: AAACTGGTAACTTGTGCTGAAGGGC, Rev: TCTGTGGTAGGTGGCGACTTGC; RUNX1-H, For: AGTTCCAGAGGGTTGAGGCAGG, Rev: TTATCAGATGACCTTGGGGTGAGC; MEF2D, For: CGGGTGCCTGTGGAGTTGG, Rev: AGGGGTCTCGGAAGCGGG; JUNB, For: GGTCCAGGGAGCAGGCGG, Rev: CCAGTGTGGTTTGCGGCG; SPI-1 URE, For: TGTGCGGTGCCTGTGGTAATG, Rev: TGCTGTGGGGGAAAACTCGG; SPI-1 ex1, For: GCTCACCCAGGGCTCCTGTAGCTC, Rev: CCATTTTGCACGCCTGTAACATCC.

**Gene Expression Analysis and Primers**

In Figure 4A, RT-PCR signals were normalized to two different housekeeping genes (*GAPDH* and *βActin*) using the ΔCT method and then the highest expressing cell line was arbitrarily set to 100 and expression in all other lines was normalized to this value.

The following *RUNX1*, *GAPDH* and *HOXA9* Taqman primer/probe sets were used for the gene expression data in Figure 3, Figure 4 and Figure 6B:

*RUNX1* 20X Taqman primer/probe set from ABI, cat# Hs00231079_m1 RUNX1

*GAPDH* 20X Taqman primer/probe set from ABI, cat# Hs03929097_g1 GAPDH

*HOXA9* Forward primer: AAAACAATGCCGAGAATGAGAGCG, Reverse primer: TGGTGTTTTGTATAGGGGGACC, FAM-TAMRA probe: CCCCATCGATCCCAATAACCCAGC

The following SYBR green primer sets were used for the gene expression data in Figure 3A, Figure 4A, Figure 5, Figure 6B and 6G and Figure S4: *MLL*, For: ACAGAAAAAAGTGGCTCCCCG, Rev: GCAAACCACCCTGGGTGTTA; *MLL-AF4* (SEM cells), For: ACAGAAAAAAGTGGCTCCCCG, Rev: TATTGCTGTCAAAGGAGGCGG; *MLL-AF4* (RS4;11 cells), For: TCAGCACTCTCTCCAATGGCAATAG, Rev: GGGGTTTGTTCACTGTCACTGTCC; *AF4-MLLder4a*, For: CAAGATCAGGCCCCTAGTGA, Rev: CCCATCTCCCACACATTTTC; *AF4-MLLder4b*, For: CAAGATCAGGCCCCTAGTGA, Rev: AGGGCTCACAACAGACTTGG; *HOXA10*, For: CGCAACCACCCCAGCCAG, Rev: TTGTCCGCCGAGTCGTAGAGG; *MEF2D*, For: CTGAGCGTGCTATGTGACTGCG, Rev: TGGAGTGGTTGAAGATGATGAGTGC; *JUNB*, For: GGTCCAGGGAGCAGGCGG, Rev: CCAGTGTGGTTTGCGGCG; *SPI-1*, For: CGGCTGGATGTTACAGGCGTG, Rev: TCGTGCGTTTGGCGTTGG; *GAPDH*, For: AACAGCGACACCCATCCTC, Rev: CATACCAGGAAATGAGCTTGACAA;

Gene expression was normalized to *GAPDH* (either Taqman or SYBR green) by the ΔCT method.

**Genomic DNA-Fragment Library**

Genomic DNA fragment libraries were prepared using the Illumina ChIPseq Library preparation Kit following the manufacturer’s instructions (Illumina, CA). Briefly 10ng of purified ChIP DNA was end repaired by conversion of overhangs into phosphorylated blunt ends with the use of T4 DNA polymerase and *E. coli* DNA polymerase I Klenow fragment. Illumina single-end adapters were ligated to the ends of the DNA fragments. Ligation products were purified on a 2% agarose gel with a size selection of 200-300bp. Fifteen PCR cycles were performed with Illumina genomic DNA primers that anneal to the ends of the adapters. The purified PCR-amplified fragment libraries were quantified with the use of the PicoGreen dsDNA Quantitation Assay with the Qubit Fluorometer (Life Technologies, CA). The size range of libraries was validated on the Agilent Technologies 2100 Bioanalyzer with the High Sensitivity DNA Kit (Agilent, CA).

**ChIP Sequencing**

After library preparation, the protocols for the Illumina Single-Read Cluster Generation Kit were used for cluster generation on the cBOT (Illumina). The targeted samples were diluted to 10 nmol and denatured with sodium hydroxide. Seven picomoles of each target-enriched sample and Phix control were loaded into separate lanes of the same flow cell, hybridized, and isothermally amplified. After linearization, blocking, and primer hybridization, sequencing was performed for 36 or 51 cycles on an Illumina GAIIx or HiSeq2000. Raw image data were converted into base calls via the Illumina pipeline CASAVA version 1.7 with default parameters. Rigorous quality control was performed with the use of data from reports generated by the Illumina pipeline.

**ChIP-Seq Data Analysis**

All 36 or 51 bp-long reads were mapped to the reference human genome sequence, hg18, using Illumina’s ELAND or BWA (Li and Durbin, 2009) aligner with the default parameters. Only reads mapping uniquely to the genome with not more than 2 mismatches were retained for further analysis. Clonal reads (i.e., reads mapping at the same genomic position and on the same strand) were collapsed into a single read. Peaks from ChIPseq data were called using the ChIPseeqer program (Giannopoulou and Elemento, 2011) with the following parameters: -t 15 -f 2 -fraglen 170. The peaks were annotated to gene bodies, defined as 2kb upstream of the TSS to 1kb downstream of the TES, and to gene promoters defined as within 2kb upstream and 2kb downstream of TSS, based on hg18 refseq genes downloaded from the UCSC Genome Browser. Regions of overlap for MLL-N and AF4-C were defined as peaks overlapping in the promoter regions (± 2kb to TSS), and for H3K79Me2 as the gene body regions (−2kb to TSS to +1kb to TES).

**Patient Data**

Gene expression microarray data from three large cohorts of patients with ALL were analyzed. The Eastern Cooperative Oncology Group (ECOG) Clinical Trial E2993 (GEO#: GSE34861) total samples = 191, BCR-ABL patients: 78, E2A-PBX1 patients: 6, MLLr patients: 25 (t(4;11): 17, other MLLr 8), Other B-ALL patients: 82, preB: 3 (Geng et al., 2012). The Children’s Oncology Group (COG) Clinical Trial P9906 ((Harvey et al., 2010), GEO#: GSE28460), clinical data downloaded from the National Cancer Institute TARGET Data Matrix at http://target.nci.nih.gov/dataMatrix/TARGET_DataMatrix.html) total samples = 207, E2A-PBX1 patients: 23, MLLr patients: 21, RUNX1-ETV6 patients: 3, Other B-ALL patients: 155, Trisomy 4 or 10 patients: 5. The St. Jude Research Hospital pediatric ALL clinical trial (Ross et al., 2003), no GEO number but raw data can be downloaded from the following site: http://www.stjuderesearch.org/site/data/ALL3/) total samples = 132, BCR-ABL patients: 15, E2A-PBX1 patients: 18, MLLr patients: 20, RUNX1-ETV6 patients: 20, Hyperdiploid patients: 17, Other B-ALL patients: 28, T-ALL patients: 14.

**Patient Gene Expression Microarray Data**

The microarray raw data was normalized using the RMA method (Bolstad et al., 2003) with Expression Console™ software (Version 1.1, Affymetrix, Santa Clara, CA) for the Affymetrix arrays HG-U133 plus2 (COG data, n = 207) or HG-U133 A and B (St Jude data, n = 132), or NimbleScan software (version 2.5, Roche NimbleGen, Madison, WI) for the NimbleGen arrays HG18 60-mer expression 385K platform (ECOG data, n = 191). The patients in each clinical trial were grouped into subtypes according to their cytogenetic features: BCR-ABL, E2A-PBX1, MLLr (MLL rearrangement), ETV6-RUNX1, or other ALLs which are negative to the above translocations. T-ALL samples were excluded from this analysis. MLL fusion partner information was available for the ECOG MLLr ALL, which were therefore further separated into MLL/AF4 (n = 17) or other MLLr (n = 8). No MLL fusion partner information was available for the COG or St Jude clinical trials, so MLLr ALL patients were treated as one group. Expression level of a gene in a sample was determined by the average of expression values from multiple probe sets on the array representing this gene. The p values of differential expression of *RUNX1*, *HOXA9*, *HOXA10* and *CDKN1B* between MLLr and other ALL subtypes were determined by two-sided Wilcoxon test. The expression values are log2 transformed so the fold change of *RUNX1* expression was calculated as 2ˆ(MLLr or t(4;11) *RUNX1* expression – other subtype expression). All downstream microarray analysis was performed using R version 2.14.0 (R Development Core Team. R: A Language and Environment for Statistical Computing. 2009; http://www.R-project.org).

**Patient Outcome Data**

In the COG P9906 ALL clinical trial (n = 207), the minimal residual disease (MRD) was assessed by flow cytometry at the end of induction therapy (day 29), as previously described (Borowitz et al., 2008), and cases were defined as MRD positive (MRD+) or MRD negative (MRD-) using a threshold of 0.01%. Among the 207 COG ALL patients, 191 patients had the MRD data available, and 17 of them were MLLr ALL. We compared *RUNX1* expression in the MRD+ and MRD- patients for all 191 ALL and for the subset of 17 MLLr ALL. P values were calculated by two-sided Wilcoxon test using R (R Development Core Team, 2009).

**Nuclear Extracts**

Nuclear Extracts were prepared using a modified Dignam protocol (Dignam et al., 1983). Briefly, 5 l of cells were grown up to a density of 1-2 X 10^6^/mL. Cell pellets were rinsed in PBS and dounce homogenized in a hypotonic buffer containing 10mM Tris-HCl pH 7.5 (pH 7.3 at 4°), 10mM KCl, 1.5mM MgCl_2_ in the presence of Roche protease inhibitors (catalog# 05056489001). Once pelleted the nuclei were resuspended in a low salt buffer (20 mM HEPES, pH 7.9 at 4°C, 25% glycerol, 1.5 mM MgCl_2_, 0.02 M KCl, 0.2 mM EDTA, 0.5 mM DTT and Roche protease inhibitors) then a high salt buffer (20 mM HEPES, pH 7.9 @ 4°C, 25% glycerol, 1.5 mM MgCl_2_, 1.2 M KCl, 0.2 mM EDTA, 0.5 mM DTT) was added, dropwise, with stirring. Nuclear extract supernatant was collected after pelleting the chromatin and quantified using a BradfordUltra (expedeon) solution.

**Immunoprecipitation Assays**

500 μg of RS4;11, SEM or CCRF-CEM nuclear extracts were diluted in a solution of 20mM Tris-Hcl pH 7.5 (pH 7.3 at 4°C), 20% Glycerol, 300mM KCl, 5mM EDTA (5mM) with protease inhibitors (Roche). Extracts were rotated overnight at 4°C with either Rabbit IgG (Millipore), AF4-N (Bethyl, A302-344A), RUNX1 (a combination of Cell Signaling, 4334 and Abcam, ab23980 antibodies) or MLL-C (Active Motif, 61295). Antibody-protein complexes were collected with a mixture of protein A and G Dynabeads (Life Technologies) and washed with 1X 20mM Tris-Hcl pH 7.5 (pH 7.3 at 4°C), 20% Glycerol, 150mM KCl, 5mM EDTA with protease inhibitors (Roche) and 1X PBS. Complexes were eluted by boiling in NuPAGE gel loading buffer (Life Technologies) for 5 min.

**Antibodies**

The following antibodies were used with the indicated techniques:

H3K4Me3 (Active Motif, 39159, ChIP and ChIPseq); H3K79Me2 (Millipore, 04-835, ChIP and ChIPseq); MLL-N (Bethyl, A300-086A, ChIP and ChIPseq); MLL-C (Bethyl, A300-374A, Western blot); MLL-C (Active Motif, 61295, IPs and ChIP), AF4-C (Abcam, ab31812, ChIP, ChIPseq and Western blot); AF4-N (Bethyl, A302-344A, ChIP, IP and Western blots); ENL (Bethyl, A302-268A, ChIP and Western blots); AF9 (Bethyl, A300-595A, ChIP and Western blots); CyclinT1 (Bethyl, A303-496A, ChIP and Western blots); aff4 (Bethyl, A302-538A, ChIP); Menin (Bethyl, A300-105A, Western blots); RUNX1 (Cell Signaling, 4334 for IPs and Western blots, Abcam, ab23980 antibodies for IPs and ChIPseq); HOXA9 (Millipore, 07-178, Western blots); GAPDH (Bethyl, A300-641A); RbBP5 (Bethyl, A300-109A, Western blots); WDR5 (Bethyl, A302-430A, Western blots); MEF2C (Cell Signaling, 5030, Western blots); LEF1 (Bethyl, A303-486, Western blots); ADAM10 (Abcam, ab1997, Western blots); ZEB2 (Bethyl, A302-474A, Western blots); SPI-1/PU.1 (Cell Signaling, 2258S, Western blots); ELL2 (Bethyl, A302-505A, Western blots); MEF2D (Bethyl, A303-521A, Western blots); JUNB (Bethyl, A302-704A, Western blots); JUND (Cell Signaling, 5000, Western blots); SPI-B (Abcam, ab42436 Western blots), βActin (Sigma, A4700).

**RNA Interference and qRT-PCR**

Briefly, 1 × 10^6^ SEM or RS4;11 cells or 5 × 10^5^ THP-1 cells in log phase growth were transfected with 1-2 μg siRNA (depending on cell type and target) using an Amaxa Nucleofector (Lonza AG) with Nucleofector Kit V, program T-020 (for SEM cells), V-001 (for THP-1 cells) or Nucleofector Kit R program T-016 (for RS4;11 cells). Cells were cultured for 48 hr and then retransfected as described above. Total RNA was extracted using Trizol (Life Technologies) 48 hr after the second transfection and cDNA was generated using SuperScript III Reverse Transcriptase (Life Technologies). qPCR analysis of the cDNA was achieved using an ABI 7500 Fast Real-Time PCR machine with the primers listed above. The following specific siRNAs were used: MLL-AF4 siRNA and control sequences used in Figure 3, siMM (scrambled control), siMARs (MLL-AF4 siRNA in RS4;11 cells) and siMA6 (MLL-AF4 siRNA in SEM cells), are from (Thomas et al., 2005). Wild-type MLL1 siRNAs: Figure 3 and Figure 6 (Dharmacon on Target Plus Smartpool, L-009914-00) versus a non-targeting smartpool control (Dharmacon On Target plus non targeting pool D001801020); *RUNX1* siRNAs: Figure 5, Figure 6 and Figure S5 (siRNA#1: Dharmacon, J003926050050) versus a non-targeting smartpool control (Dharmacon On Target plus non targeting pool D001801020), Figure 5 and Figure 6 siRNA#2 (Life Technologies stealth siRNA, sequence from Suzuki et al., 2009) versus a non targeting control (Life Technologies, Stealth negative Control Medium GC, 12935). In Figure S5E–5SG two different AF4-MLL siRNAs were used to transfect SEM cells: AF4-MLL siRNA-K from Kumar et al. (2011) and AF4-MLL siRNA#10 (CAGTTGAGGAGGATTGTGA). Transfected cells were compared to cells treated with a siRNA universal negative control (SIGMA SIC002).

## Accession Numbers

The Gene Expression Omnibus accession number for the RUNX1 ChIP-seq data reported in this paper is GSE42075.

## Supplemental Information

Table S1. MLL-AF4 ChIP-Seq Target Genes in RS4;11 and SEM Cells, Related to Figure 1

Document S1. Tables S2–S4

Table S5. RUNX1 ChIP-Seq Target Genes, Related to Figure 6

Document S2. Article plus Supplemental Information
