# Supplementary material for: RUNX1 Is a Key Target in t(4;11) Leukemias that Contributes to Gene Activation through an AF4-MLL Complex Interaction
Source: Cell Rep. 2013 Jan 31;3(1):116–27. doi: 10.1016/j.celrep.2012.12.016 (PMC3607232; doi:10.1016/j.celrep.2012.12.016)
Supplement: Document S1. Tables S2–S4 [file mmc2.pdf]

Table S2. P-Values in Three B-ALL Clinical Trials, Related to Figure 2 and Figure S2

St Jude ALL pvalues		BCR-ABL (n=15)	E2A-PBX1 (n=18)	ETV6-RUNX1 (n=20)	Others (n=28)	preB
RUNX1	MLLr (n=20)	0.006	n.s.	n.s.	0.002	N/A
HOXA9	MLLr (n=20)	0.0002	0.0001	4.51E-05	3.39E-05	N/A
HOXA10	MLLr (n=20)	0.0008	0.0012	2.28-04	2.15E-02	N/A
CDKN1B	MLLr (n=20)	0.0002	1.61E-07	2.02E-09	4.25E-08	N/A
COG ALL P9906 pvalues		BCR-ABL	E2A-PBX1 (n=23)	ETV6-RUNX1 (n=3)	Others (n=155)	preB
RUNX1	MLLr (n=21)	N/A	0.004	n.s.	0.0005	N/A
HOXA9	MLLr (n=21)	N/A	2.04E-07	n.s.	3.70E-10	N/A
HOXA10	MLLr (n=21)	N/A	9.76E-07	n.s.	2.38E-09	N/A
CDKN1B	MLLr (n=21)	N/A	0.019	0.001	0.005	N/A
ECOG ALL E2993 pvalues		BCR-ABL (n=78)	E2A-PBX1 (n=6)	MLLr (n=8)	Others (n=82)	preB (n=3)
RUNX1	t(4;11) (n=17)	0.004	n.s.	0.037	0.041	0.028
HOXA9	t(4;11) (n=17)	1.96E-10	0.0004	n.s.	2.02E-10	0.008
HOXA10	t(4;11) (n=17)	1.62E-10	2.00E-05	n.s.	1.32E-10	0.0018
CDKN1B	t(4;11)(n=17)	2.05E-02	n.s.	n.s.	5.00E-03	0.007

n.s.; non significant, p>0.05

Table S3. Average Expression of *RUNX1* in Three B-ALL Patient Cohorts, Related to Figure 2

RUNX1 average expression values	MLLr avg.	BCR-ABL avg.	E2A-PBX1 avg.	ETV6-RUNX1 avg.	Others avg.	preB avg.	t(4;11) avg.	Other MLLr avg.
St Jude	4.659	4.433	4.647	4.568	4.447	N/A	N/A	N/A
COG ALL P9906	8.509	N/A	7.982	7.423	8.014	N/A	N/A	N/A
ECOG ALL E2993	9.024	8.573	8.616	N/A	8.791	7.961	9.174	8.703

Table S4. Fold Change of *RUNX1* Gene Expression in Three B-ALL Patient Cohorts, Related to Figure 2

RUNX1 average expression fold change	BCR-ABL avg. fold change	E2A-PBX1 avg. fold change	ETV6-RUNX1 avg. fold change	Others avg. fold change	preB avg. fold change	Other MLLr avg. fold change
St Jude (MLLr vs. others)	1.169647<br>p=0.006	1.008513<br>p= n.s.	1.065644<br>p= n.s.	1.158339<br>p=0.002	N/A	N/A
COG P9906 (MLLr vs. others)	N/A	1.441242<br>p=0.004	**2.122593**<br>**p=0.08**	1.409682<br>p=0.0005	N/A	N/A
ECOG E2993<br>(t-4;11 vs. others)	1.516827<br>p=0.004	1.472198<br>p= n.s.	N/A	1.30465<br>p=0.041	**2.318857**<br>**p=0.028**	1.386325<br>p=0.037

n.s.; non significant, p>0.05
